# Amyand’s Hernia: A Rare Case Study of Perforated Appendicitis in an Inguinal Hernia

**DOI:** 10.7759/cureus.56898

**Published:** 2024-03-25

**Authors:** Laura Chagam, Raahi Modi, Frank Toub

**Affiliations:** 1 Medicine, Lake Erie College of Osteopathic Medicine, Bradenton, USA; 2 General Surgery, AdventHealth New Smyrna Beach, New Smyrna Beach, USA

**Keywords:** enterocutaneous fistula, periappendiceal abscess, perforated appendicitis, inguinal hernia, amyand’s hernia

## Abstract

Amyand’s hernia is defined as the presence of an appendix contained within an inguinal hernia. An inguinal hernia is the protrusion of a small or large bowel through a peritoneal defect of the groin. In rare cases, the appendix can become incarcerated or strangulated within the hernia, cutting off the blood supply to the organ. If incarcerated, the appendix is at risk for strangulation, which can lead to rupture and cause worsening of symptoms and/or collapse. We report a case of a 76-year-old male with a history of inguinal hernia repair 30 years prior, who presented with 30 days of intermittent right lower quadrant pain and unintentional weight loss. He required emergent treatment and management. This case underscores the challenge of diagnosing concurrent inguinal hernia and appendicitis and places an emphasis on timely intervention. Treatment options vary based on the appendix’s apparent condition within the hernia sac. Despite the complications, including additional drainage site and enterocutaneous fistula, the patient was discharged in stable condition. This case contributes insights into managing complex inguinal pathologies, particularly in the geriatric population.

## Introduction

An inguinal hernia arises when an iatrogenic weakness in the abdominal muscular wall allows a portion of an organ or its fascia to protrude into the groin. Inguinal hernias are usually found superior to the inguinal ligament. Direct inguinal hernias are anteromedial to the inferior epigastric vessels, while indirect hernias, being the most common, protrude posterolateral to the vessels [1]. When an organ is contained in the groin and cannot be reduced, this is known as an incarcerated hernia. Incarcerated hernias can become inflamed and edematous. A strangulated hernia is a fatal condition that occurs when the blood supply to the incarcerated portion of the bowel is compromised. Strangulated hernias can have compromised venous outflow, and reduced arterial inflow, and are at risk for necrosis. Hernia recurrence can be due to age-related weakening of the abdominal wall, occupation-induced strain, and poor surgical technique [2].

Amyand’s hernia is defined as the presence of an appendix within an inguinal hernia. Amyand’s hernia was originally described by Claudius Amyand, who performed the first successful appendectomy on December 6, 1735, on an 11-year-old boy, Hanvil Anderson, who had a right inguinal hernia. Hanvil’s inguinal hernia contained an infected appendix that had been perforated by a swallowed needle [3,4].

The incidence of Amyand’s hernia ranges from 0.19% to 1.7% of reported hernia cases. It is three times more likely to be diagnosed in children than in adults, due to the patency of processus vaginalis [5]. While the incidence of this type of hernia is rare, an appendix within an inguinal hernia can become incarcerated and can lead to strangulation and perforation. The incidence of appendicitis, inflammation of the appendix, contained within an inguinal hernia is even rarer, with a rate of 0.07% to 0.13% [6]. We present a case of a patient with an inguinal hernia that was confounded by concurrent perforated appendicitis (Losanoff and Basson classification type 3).

## Case presentation

A 76-year-old male with a past medical history of left inguinal hernia repair 30 years ago, essential tremor, gastroesophageal reflux disease, hyperlipidemia, and hypertension was initially evaluated in the surgery clinic for right groin pain and had an outpatient computed tomography (CT) of the abdomen and pelvis. The CT revealed a right inguinal hernia containing the appendix with abnormal enhancement, fluid up to 4.5 cm, and surrounding inflammatory changes. He presented to the emergency department after being referred by General Surgery for evaluation of abdominal pain. He reported having intermittent abdominal pain and unintentional weight loss for a month prior. The pain worsened with movement and palpation. He reported not having a bowel movement for several days but attributed it to decreased oral intake. He denied any fever, chills, chest pain, palpitations, shortness of breath, dizziness, nausea, vomiting, and diarrhea.

**Figure 1:**
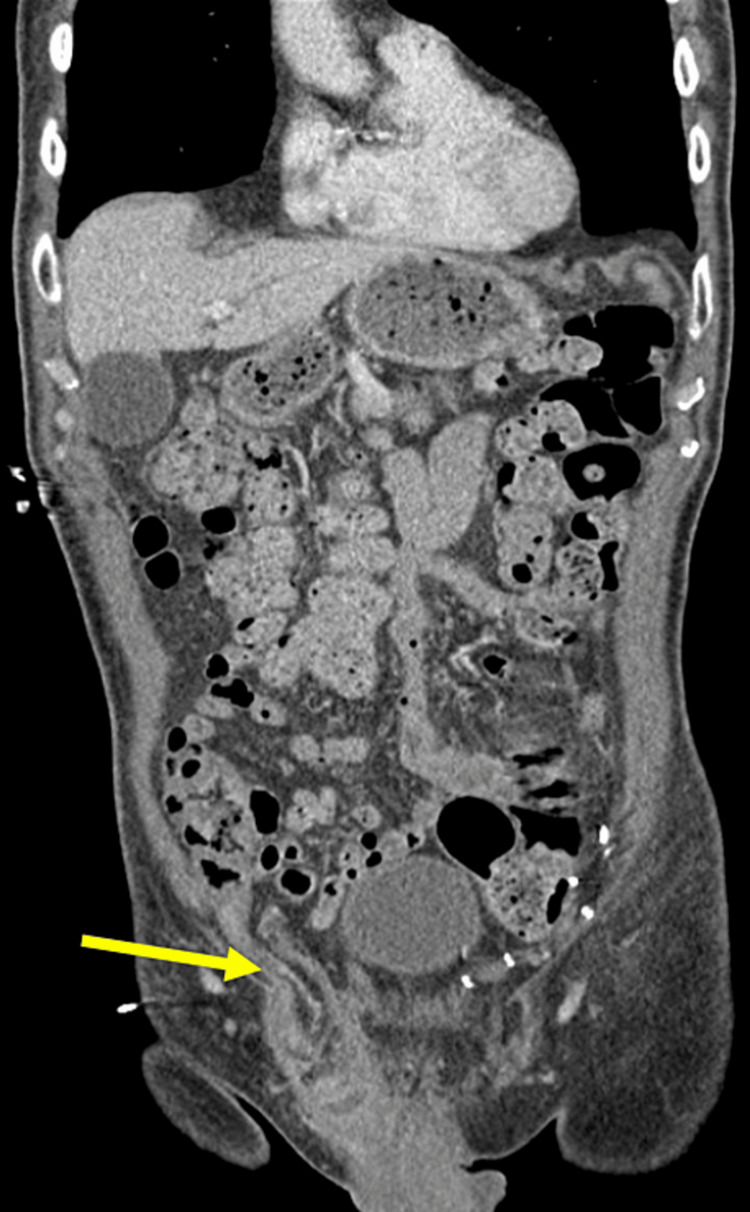
CT abdomen pelvis with IV contrast. Thickened appendix extends into the right inguinal hernia. Interval improvement in the size of fluid collection in the right inguinal hernia with residual collection after placement of a percutaneous pigtail catheter.

**Figure 2:**
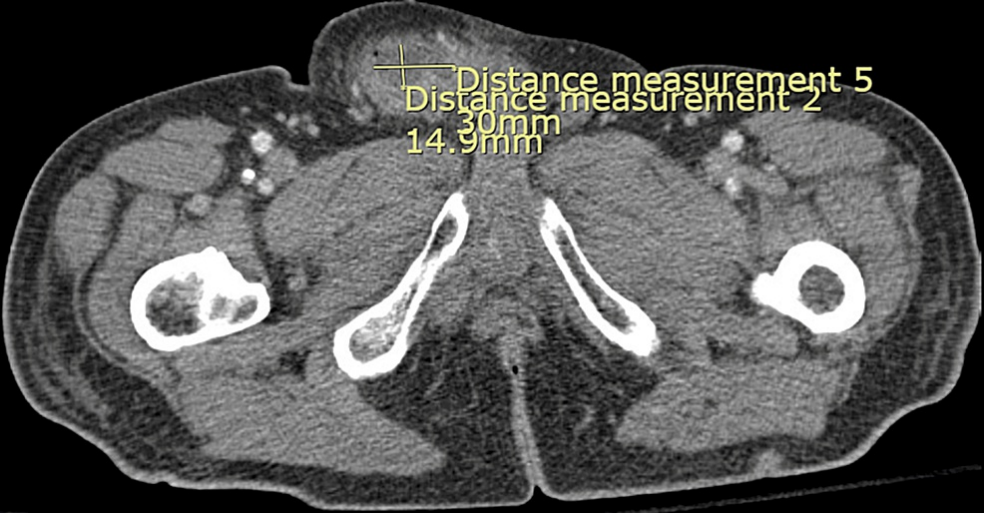
Fluid collection in the right inguinal hernia with residual small collection after drainage measuring approximately 3.1 x 1.5 x 4.3 cm.

Initial vital signs on presentation to the emergency department were as follows: blood pressure of 120/67, pulse rate of 79 bpm, temperature of 100℉, respiratory rate of 20 breaths per minute, 98% oxygenation on room air, and BMI of 19.53 kg/m^2^. He appeared frail and deconditioned. Laboratory tests included a complete blood count and comprehensive metabolic panel. The results of the tests included leukocytosis with left shift, mild hyponatremia, hypochloremia, and mildly elevated BUN (Table 1). C-reactive protein was elevated at 76.20 (reference: <5.00 mg/L). An interventional radiologist (IR) performed CT-guided drainage of the periappendiceal abscess with catheter placement (Figures 1, 2). A body fluid culture was taken from the appendix after the drain placement and showed heavy growth of* Escherichia coli* and moderate *Enterobacter cloacae*. The susceptibility screen resulted in *E. coli* being resistant to ampicillin and cefazolin. The patient was treated with IV piperacillin/tazobactam 4.5 g every eight hours at 25 mL/hour for four hours and IV fluconazole 200 mg every 24 hours at 100 mL/hour over 60 minutes for three days.

**Table 1:** Laboratory values. All other values are within normal limits unless stated otherwise. ^1^Hemoglobin A1C was 5.5% considered to be non-diabetic range.

	Reference Range	Laboratory value
WBC	3.60 - 10.00 10^3^/uL	16.45
Hemoglobin	14.0 - 18.0 g/dL	12.9
RDW	11.5 - 14.5%	14.6
Neutrophils	40.0 - 85.0%	89.7
Lymphocytes	20.0 - 40.0%	5.0
Sodium	136 - 145 mmol/L	134
Chloride	98 - 107 mmol/L	95
BUN	8.0 - 23.0 mg/dL	24.0
Glucose^1^	70 - 99 mg/dL	158

**Figure 3:**
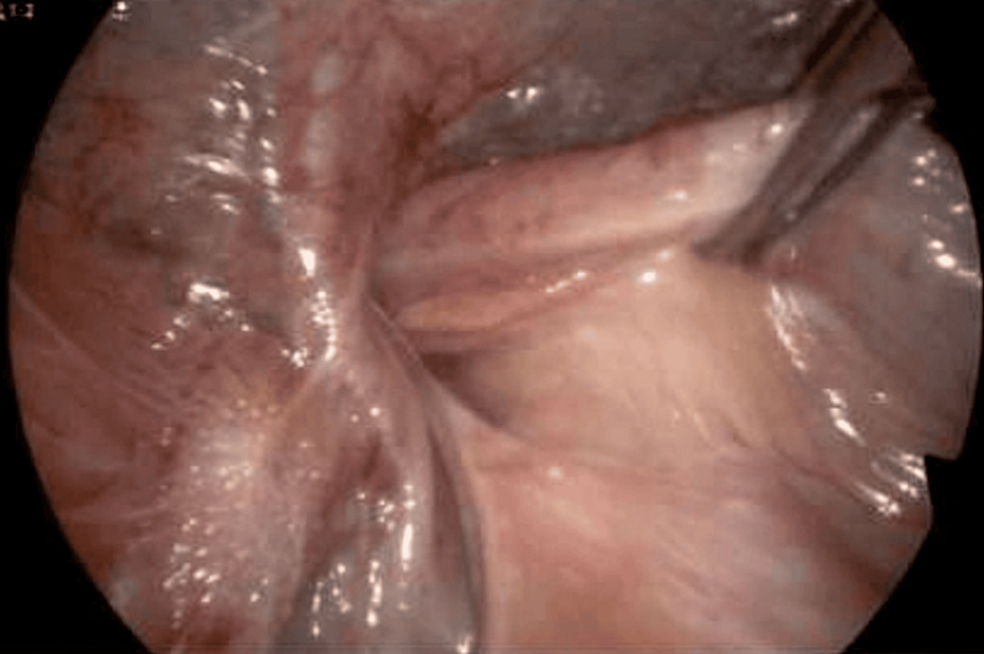
Laparoscopic image. The site of the inguinal hernia.

**Figure 4:**
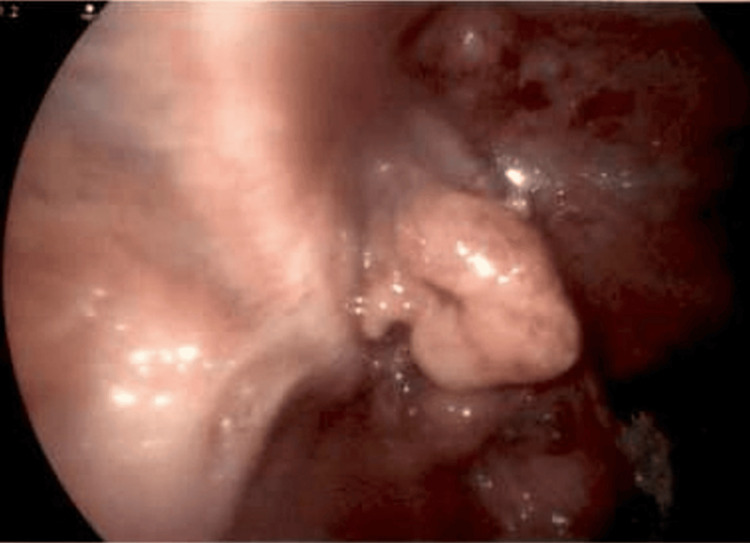
Laparoscopic image. Appendix inverted out of the inguinal hernia canal for visualization.

The patient was treated and monitored for six days and was switched to metronidazole 500 mg tablet twice daily for 14 days upon discharge to a skilled nursing facility. He was advised to follow up with general surgery. The patient’s drain fell out within 16 days after discharge, and he was brought back to the emergency room due to increased pain. His drainage site was erythematous, edematous, and leaking fluid. Repeat CT of the abdomen and pelvis with IV contrast showed a right inguinal hernia and an increase in the size of the abscess to 3.6 x 2.1 x 6.7 cm. Additionally, a 9-mm ureterovesical junction (UVJ) obstruction stone, which the urologist confirmed to be a phlebolith with moderate constipation. He received IV piperacillin/tazobactam 4.5 g and IV vancomycin 1 g for one dose and the drain was replaced during this visit. The culture from the abscess was positive for *Klebsiella ESBL*. He was placed on IV meropenem 1g NaCl 50 mL reconstituted solution by infectious disease. Four days after drain placement, the patient had about 150 cc of purulent drainage from a new site due to forming an enterocutaneous fistula. The patient was scheduled for laparoscopic appendectomy, lysis of adhesions, and drainage of the chronic fistula (Figures 3, 4). He tolerated the procedure well and returned to recovery in stable condition. Five days post-operation, general surgery recommended that the patient was stable for discharge to rehabilitation if the hernia mesh did not become infected. The patient was discharged to a skilled nursing facility and continued on meropenem-NaCl 500 mg injection and oxycodone 5 mg.

## Discussion

Amyand’s hernia is usually an incidental finding on imaging or during surgery. While the pathophysiology of Amyand’s hernia is unclear, the proposed theories include congenital laxity of the right colon or the co-existence of a fibrous connection between the appendix, testes, and a patent vaginal process [7]. It presents as sudden onset epigastric pain with localized tenderness in the right lower quadrant and a tender, irreducible mass in the inguinal region. A preoperative diagnosis is made with a CT scan of the abdomen and pelvis along with clinical symptoms [8]. Inguinal hernias are diagnosed clinically, but a CT is often utilized when the patient is being evaluated for acute abdomen or further testing for an abdominal hernia. A CT is usually ordered to rule out serious pathology, such as a carcinoma or hemoperitoneum, and can increase the specificity in diagnosing an Amyand’s hernia by 90% [9]. Ultrasound is a cheaper option that can also be used to diagnose hernias, but it is relatively unreliable and dependent on the technical skills of the operator. Once diagnosed, laparoscopic appendectomy with primary hernia repair is the treatment of choice. If the appendix is inflamed or an abscess has formed, the patient should be given appropriate antibiotics. A drain should be placed until the abscess resolves before proceeding with the repair. The procedure can be staged or completed on a single visit depending on severity [4].

The current treatment algorithm for Amyand’s hernia is based on the classification and condition of the appendix contained within the hernia sac. Losanoff and Basson created a simplified classification scale to manage various types of Amyand’s hernia (Table 2). A Type 1 hernia is a normal appendix in an inguinal hernia, which is managed by a reduction and mesh repair. Types 2-4 have acute appendicitis contained within the hernia sac. Type 2 is an inflamed non-perforated appendix. Type 3 is a perforated appendix and type 4 is complicated with an abdominal pathology [10]. 

**Table 2:** Losanoff and Basson classification of Amyand’s hernia

Classification	Description	Surgical management
Type 1	Normal appendix in an inguinal hernia	Hernia reduction, mesh repair
Type 2	Acute appendix in an inguinal hernia, without abdominal sepsis	Appendectomy, primary repair of hernia without mesh
Type 3	Acute appendix in an inguinal hernia, with abdominal wall or peritoneal sepsis	Laparotomy, appendectomy, primary repair without mesh
Type 4	Acute appendix in an inguinal hernia, with abdominal pathology	Manage as Type 1-3, investigate pathology as needed

Our patient had a Type 3 Amyand’s hernia and underwent a laparoscopic appendectomy with drainage of chronic fistula, lysis of multiple adhesions, and primary hernia repair with mesh. Hernia mesh, also known as surgical mesh, is used to support damaged tissue around a hernia while it heals. During laparoscopic surgery, the mesh is placed in the peritoneal space using tacks, glues, and sutures through a small incision in the abdomen [11]. Compared to open repair surgery, laparoscopic surgery is minimally invasive and is associated with fewer complications and less pain in early postoperative stages [12]. Laparoscopic surgery reduces injury and ischemia to the peritoneum, reduces exposure to foreign material, and prevents drying of the peritoneal surface, which contributes to adhesion formation. Adhesions, which contribute to abdominal pain and complications during subsequent surgeries, result from scar tissue between the abdominal wall, intestines, and other nearby structures [13]. Common complications that arise as a result of the introduction of mesh include mesh migration, chronic pain, infection, and seroma formation [14].

## Conclusions

This case showcases the simultaneous presentation of two distinct medical conditions, acute appendicitis with perforation and right inguinal hernia. Either diagnosis could independently account for the right lower quadrant pain observed in our patient. The presence of a right groin bulge raised significant concerns about an inguinal hernia. The additional symptoms suggested a more complex situation, ultimately revealed as an acute case of appendicitis with perforation within the hernia. The abdominal CT along with cultures from the appendix allowed for a proper diagnosis. Due to the acuity of the presentation, the patient underwent interventional radiology drain installation for the periappendiceal abscess. The patient was treated with IV metronidazole and recommended to continue tablets at a skilled nursing facility. He was readmitted after 16 days due to drain displacement and leaking. Following drain replacement, he underwent a laparoscopic appendectomy with drainage of the chronic fistula and lysis of adhesions. Clinically stable and having improved symptomatically, the patient was discharged five days post-operation. While a hernia at age 76 might not require immediate attention, the severity of his symptoms pointed towards a more urgent problem and ultimate diagnosis of a perforated appendix.
